# The YTH Domain Family of N6-Methyladenosine “Readers” in the Diagnosis and Prognosis of Colonic Adenocarcinoma

**DOI:** 10.1155/2020/9502560

**Published:** 2020-05-30

**Authors:** Dian Xu, Jun Shao, Huan Song, Jianming Wang

**Affiliations:** ^1^Department of Epidemiology, Center for Global Health, School of Public Health, Nanjing Medical University, Nanjing 211166, China; ^2^Department of Ultrasound Diagnosis, The First People's Hospital of Kunshan, Suzhou 215300, China

## Abstract

To profile the landscape of methylation N^6^ adenosine (m^6^A) RNA regulators in colonic adenocarcinoma (COAD) and to explore potential diagnostic and prognostic biomarkers, we assessed the differential expression patterns of m^6^A RNA methylation regulators between 418 COAD patients and 41 controls based on profiling from The Cancer Genome Atlas (TCGA) database. We plotted the receiver operating characteristic (ROC) curves and calculated the area under the curve (AUC) values to estimate the discrimination ability. The relationship between the expression of m^6^A RNA methylation regulators and clinicopathological characteristics was explored. Kaplan-Meier plotter, log-rank test, and Cox regression were used and a nomogram was created to explore the prognostic significance of m^6^A-related genes in overall survival at the mRNA level. Pathway analysis was performed by gene set enrichment analysis (GSEA) using TCGA dataset, and a coexpression network was built based on the STRING database. We observed that YTHDF1, METTL3, and KIAA1429 were significantly upregulated, while YTHDF3, YTHDC2, METTL14, and ALKBH5 were significantly downregulated in COAD samples compared to normal samples. YTHDF1 had the highest diagnostic value. Low expression of YTHDF3 predicted a poor survival rate in COAD patients. YTHDC2 was related to sex and showed a downward trend as clinical stage increased. Our results indicate that the YT521-B homology (YTH) domain family (“readers”), especially YTHDF1, YTHDF3, and YTHDC2, might play a significant role in the detection, progression, and prognosis of COAD, indicating that they are promising cancer biomarkers.

## 1. Introduction

Colon cancer is the third most common cancer and the second leading cause of cancer-related death worldwide [1]. Colonic adenocarcinoma (COAD) is the typical type that accounts for 98% of new cases [2]. Despite advances in diagnosis and treatment [3], the prognosis of patients remains poor. Chemotherapy has shown significant value, but surgery is the only method of curative treatment. Thus, it is crucial to discover novel diagnostic and prognostic biomarkers for COAD.

Epigenetic modification has gained increasing attention in research on carcinogenesis and progression. Methylation is a common epigenetic trait and a simple biochemical process—it is the transfer of four atoms, one carbon atom and three hydrogen atoms (CH3), from one substance to another [4]. The N6-methyladenosine (m^6^A) modification of mRNA/lncRNA is the most common RNA methylation, and it can regulate cell fate determination, self-renewal, and cancer development, indicating it as a promising therapeutic target for cancer [5]. Three major types of enzymes participate in m^6^A methylation: “writers,” “erasers,” and “readers.” “Writers” mainly include METTL3, METTL14, KIAA1429, and WTAP. METTL3 and METTL14 form a structure with other enzymes (e.g., WTAP) to methylate the sixth N of adenosine during transcription from DNA to RNA [6]. The m^6^A-modified bases can be demethylated under the action of "erasers" enzymes, such as FTO and ALKBH5, making RNA methylation a reversible reaction [7]. These methylated RNA base sites finally require specific enzymes ("readers") to recognize. YTHDF1, YTHDF2, and YTHDF3 are primary members of the "readers" family, devoted to recognizing bases that undergo m^6^A methylation, participating in downstream translation, mRNA degradation, and accelerating the rate at which mRNA leaves the nucleus [8].

Aberrant expression of m^6^A writers, erasers, or readers can lead to the deregulation of m^6^A modification, thus triggering the abnormal translation of specific mRNAs and promoting or inhibiting carcinogenesis and cancer progression [9]. Accumulative evidence has supported the correlation between aberrant cellular m^6^A and human cancers [10]. For example, the overexpression of YTHDF1 is related to poor prognosis in patients with liver cancer [11]; downregulation of ALKBH5 in MDA-MB-231 human breast cancer cells can lead to a decrease in the number of breast cancer stem cells, resulting in a significant reduction in tumorigenic capacity [12].

Although traditional prognostic factors for COAD, such as age, tumor stage, surgical margins, and tumor grade, have produced significant improvements in predicting patient clinical outcomes [13], their limitations in distinguishing the prognostic value of molecular heterogeneity should not be ignored. With deepening research on RNA methylation regulators, the roles of m^6^A in diagnosis and treatment have gradually become valued. This study is aimed at profiling the landscape of m^6^A RNA methylation regulators in COAD and at exploring their potential value as diagnostic and prognostic biomarkers.

## 2. Materials and Methods

### 2.1. Data Processing

We downloaded the expression data and clinicopathological data of 418 COAD patients and 41 controls after the deletion of missing records from TCGA database (https://tcga-data.nci.nih.gov/tcga/) [14]. Age at diagnosis, sex, follow-up days, and clinical data were retrospectively extracted. All patients were staged using the 2009 TNM classification system recommended by the American Joint Committee on Cancer. We used the edgeR package to normalize the expression data.

### 2.2. Portraying the Landscape of m^6^A RNA Methylation Regulator Expression

The expression levels of six m^6^A RNA methylation regulators between tumor and normal tissues were compared using the *t*-test. The expression pattern of m^6^A RNA methylation regulators in COAD samples was denoted by the Pearson correlation matrix, and a correlation coefficient (*r*) was calculated. We plotted the ROC curve and calculated the area under the curve (AUC) to estimate the discrimination ability. The m^6^A RNA methylation regulators and clinicopathological features were analyzed using the *t*-test or one-way ANOVA.

### 2.3. Survival Analysis

A total of 418 patients were followed, with a maximum follow-up period of 4502 days. The median survival time was 670.5 days. All patients had no chemotherapy history and underwent the same type of radical surgery and postoperative adjuvant chemotherapy. The overall survival (OS) was calculated from the date of diagnosis to the date of death. We categorized the expression of m^6^A-related mRNA into two groups using the lower quartile as the cutoff point. Kaplan-Meier analysis was used to portray the survival curves of m^6^A RNA methylation regulators. The log-rank test was used to compare the survival distributions between groups. We performed univariate and multivariate analyses to determine the independent prognostic factors using the Cox proportional hazard model. A nomogram was generated based on the significant prognostic factors in the Cox regression model to predict the 1-year, 3-year, and 5-year survival of COAD patients.

### 2.4. Biological Function Analysis

We further used gene data in TCGA database to search for pathways related to YTHDF1, YTHDF3, and YTHDC2 by gene set enrichment analysis (GSEA) using GSEA v2.2.2 software (http://www.broadinstitute.org/gsea). GSEA revealed significant differences in the enrichment of genes in the MSigDB Collection (c2.cp.kegg. v7.2. symbols) [15]. The high- and low-expression phenotype groups were divided according to the lower quartile expression level of candidate genes. Gene set permutations were performed 1000 times for each analysis. The expression of selected genes was used as a phenotype label. We selected the most significantly enriched signal pathway using the criterion of NOM *P* value < 0.05, FDR *q* value < 0.25, and high normalized enrichment score (NES). The Retrieval of Interacting Genes (STRING v10) (http://string-db.org/) tool was used to analyze the interactive relationships to construct a protein-protein interaction (PPI) network. Experimentally validated interactions with a combined score > 0.4 were regarded as significant. We also selected the YTHDF1, YTHDF3, and YTHDC2 modules and constructed a PPI network of these selected m^6^A RNA regulators.

### 2.5. Statistical Analysis

A comparison of normalized data between two groups was conducted using *t*-test or Mann-Whitney *U* test by GraphPad Prism 7 (GraphPad Software, La Jolla, CA). Comparisons among multiple groups were performed using one-way ANOVA. Other analyses were visualized using R software (v.3.4.3). The significant level was set at 0.05.

## 3. Results

### 3.1. The Landscape of m^6^A RNA Methylation Regulators in COAD

**Figure 1:**
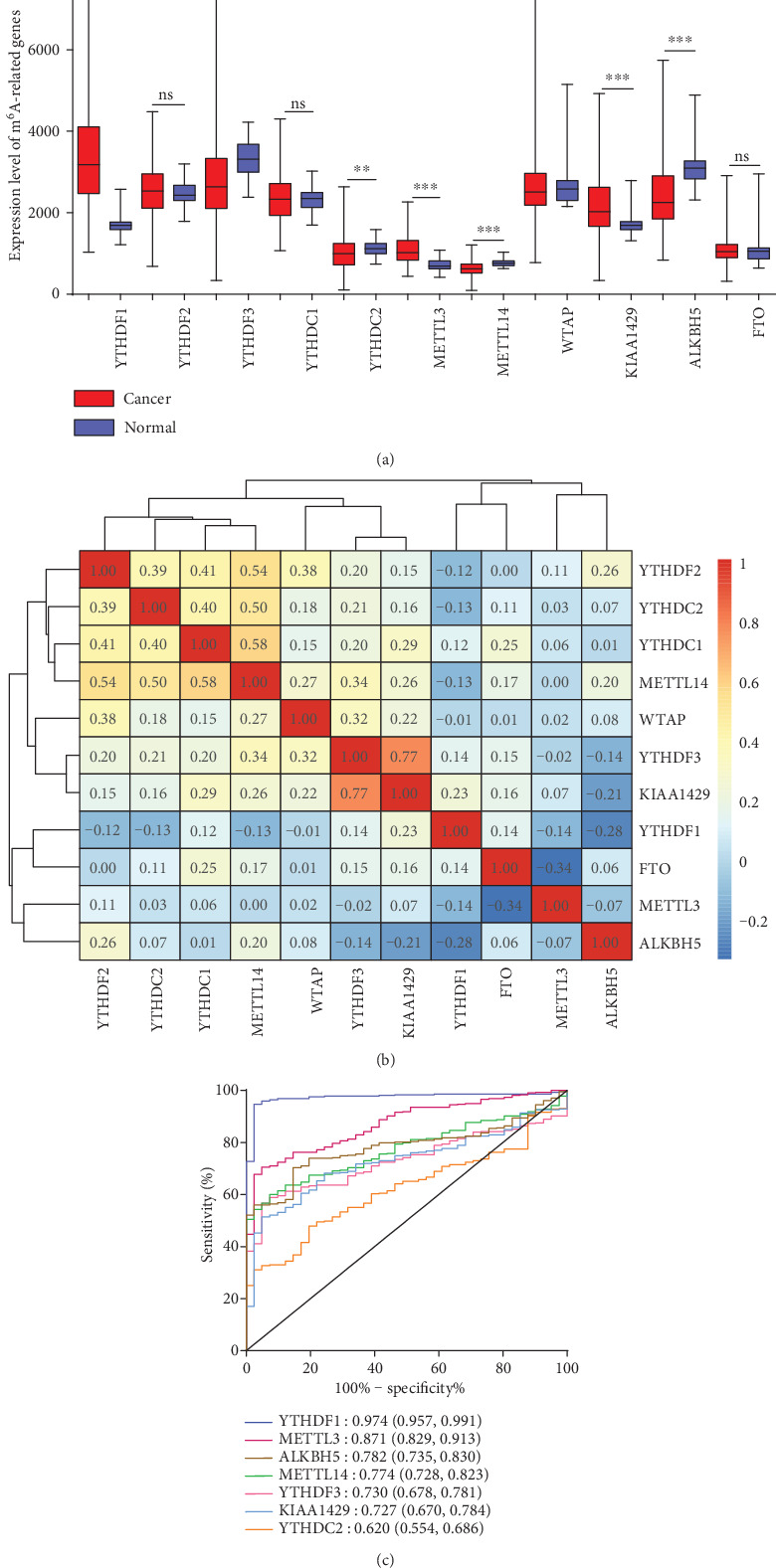
The landscape of m^6^A RNA methylation regulators in COAD. (a) The expression level of m^6^A RNA methylation regulators in cancer and normal tissues. ns: *P* ≥ 0.05; ∗: *P* < 0.05; ∗∗: *P* < 0.01; ∗∗∗: *P* < 0.001. (b) The Pearson correlation matrix among 11 m^6^A modification regulators in COAD. The correlation coefficient is shown in each grid with different colors. The redder the color is, the higher the correlation coefficient; the bluer the color is, the lower the correlation coefficient. (c) ROC curves of seven differentially expressed m^6^A RNA methylation regulators. Each curve represents a candidate gene, and the AUC is shown at the bottom right.

A total of 418 COAD cases and 41 normal controls were included in the analysis. We compared the expression of 11 m^6^A RNA methylation regulators (YTHDF1, YTHDF2, YTHDF3, YTHDC1, YTHDC2, METTL3, METTL14, WTAP, KIAA1429, ALKBH5, and FTO). As shown in Figure 1(a), YTHDF1 (*P* < 0.001), METTL3 (*P* < 0.001), and KIAA1429 (*P* < 0.001) were significantly upregulated, while YTHDF3 (*P* < 0.001), YTHDC2 (*P* = 0.020), METTL14 (*P* < 0.001), and ALKBH5 (*P* < 0.001) were significantly downregulated in COAD samples compared to normal samples. No significant difference was observed for YTHDF2 (*P* = 0.377), YTHDC1 (*P* = 0.679), WTAP (*P* = 0.472), and FTO (*P* = 0.214).

Figure 1(b) shows the clustering of different genes and their correlation coefficients in COAD. YTHDF3 and KIAA1429 were the most positively correlated (*r* = 0.77). We also found a positive correlation in the cluster of METTL14, YTHDC1, YTHDC2, and YTHDF2, with correlation coefficients ranging from 0.4 to 0.6.

The ROC curves of 7 differentially expressed genes (YTHDF1, YTHDF3, YTHDC2, METTL3, METTL14, KIAA1429, and ALKBH5) are plotted in Figure 1(c). YTHDF1 had the highest AUC value (AUC: 0.974; 95% CI: 0.957-0.991), demonstrating the high sensitivity and specificity of YTHDF1 for COAD diagnosis.

### 3.2. m^6^A RNA Methylation Regulators and Clinicopathological Features

**Figure 2:**
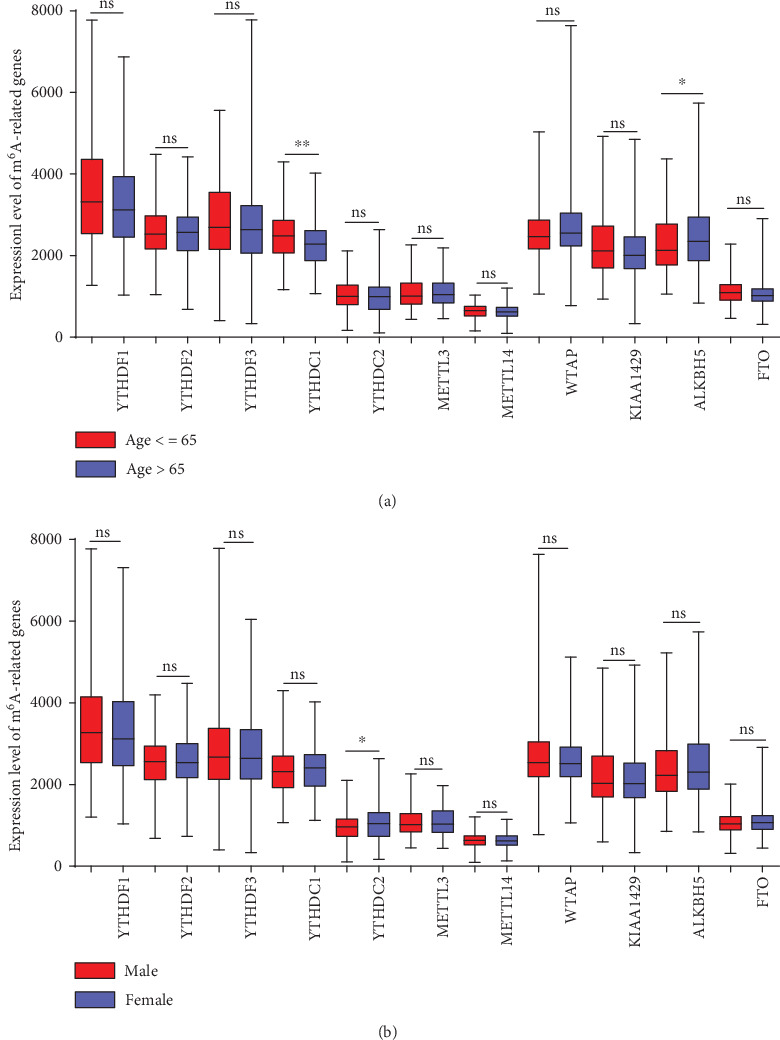
The relationship between m^6^A RNA methylation regulator expression and clinical features of COAD. (a) Expression levels stratified by age group. (b) Expression levels stratified by sex. ∗: *P* < 0.05; ∗∗: *P* < 0.01; ns: *P* ≥ 0.05.

**Figure 3:**
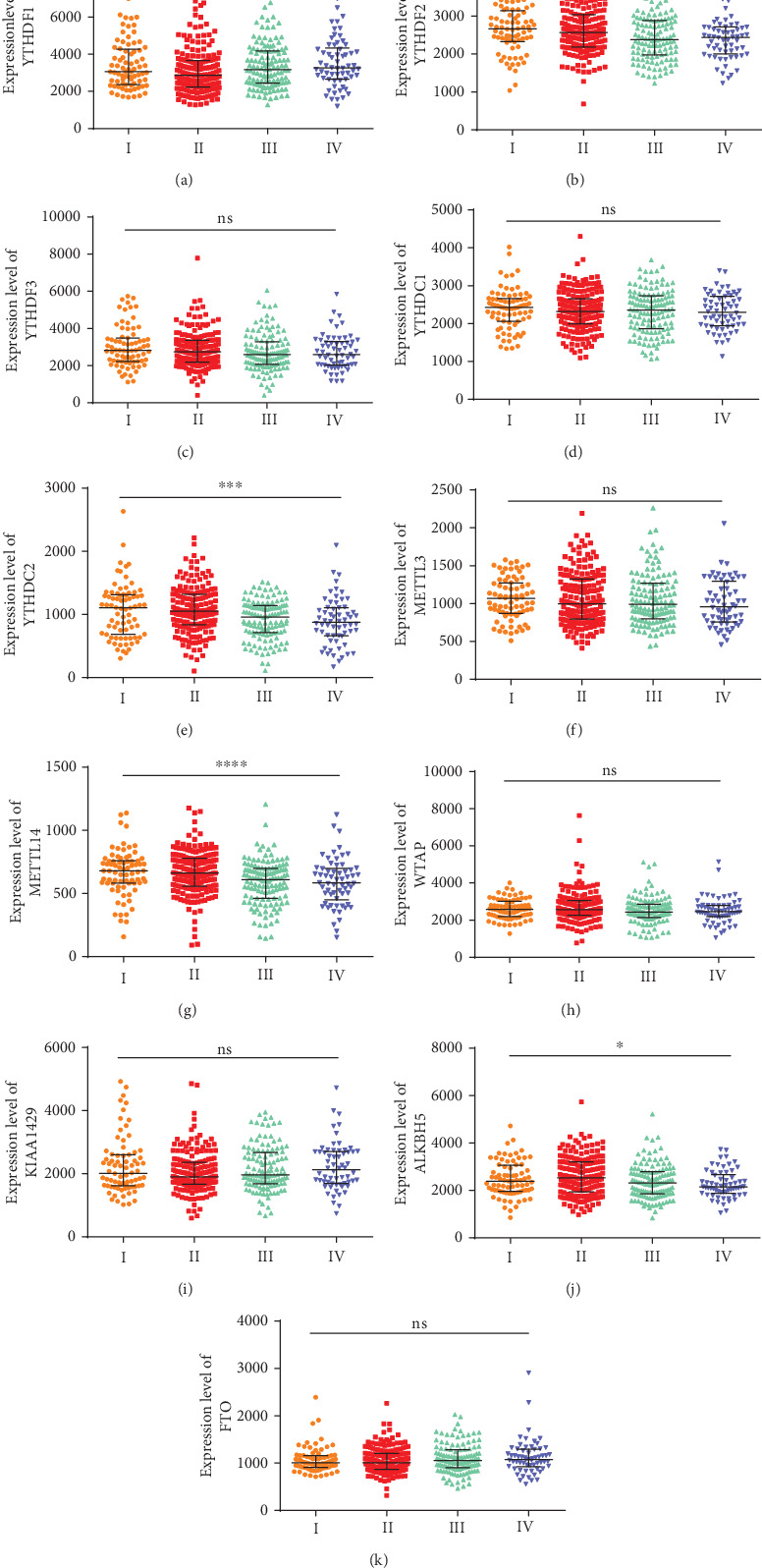
Correlation of the expression levels of m^6^A RNA methylation regulators with clinical stages. (a) YTHDF1; (b) YTHDF2; (c) YTHDF3; (d) YTHDC1; (e) YTHDC2; (f) METTL3; (g) METTL14; (h) WTAP; (i) KIAA1429; (j) ALKBH5; (k) FTO. The horizontal axis shows the clinical stages (I-IV). ns: *P* ≥ 0.05; ∗: *P* < 0.05; ∗∗: *P* < 0.01; ∗∗∗: *P* < 0.001; ∗∗∗∗: *P* < 0.0001.

Compared to younger patients, patients older than 65 years had a lower expression level of YTHDC1 (*P* < 0.0001) and a higher expression level of ALKBH5 (*P* = 0.011) (Figure 2(a)). YTHDC2 (*P* = 0.028) was highly expressed in female patients (Figure 2(b)). Clinical stage was associated with YTHDF1 (Figure 3(a), *P* = 0.028), YTHDC2 (Figure 3(e), *P* < 0.001), METTL14 (Figure 3(g), *P* < 0.0001), and ALKBH5 (Figure 3(j), *P* = 0.015) expression. YTHDC2 and METTL14 showed a significant downward trend with increasing clinical stage.

### 3.3. Prognostic Value of m^6^A RNA Methylation Regulators

**Figure 4:**
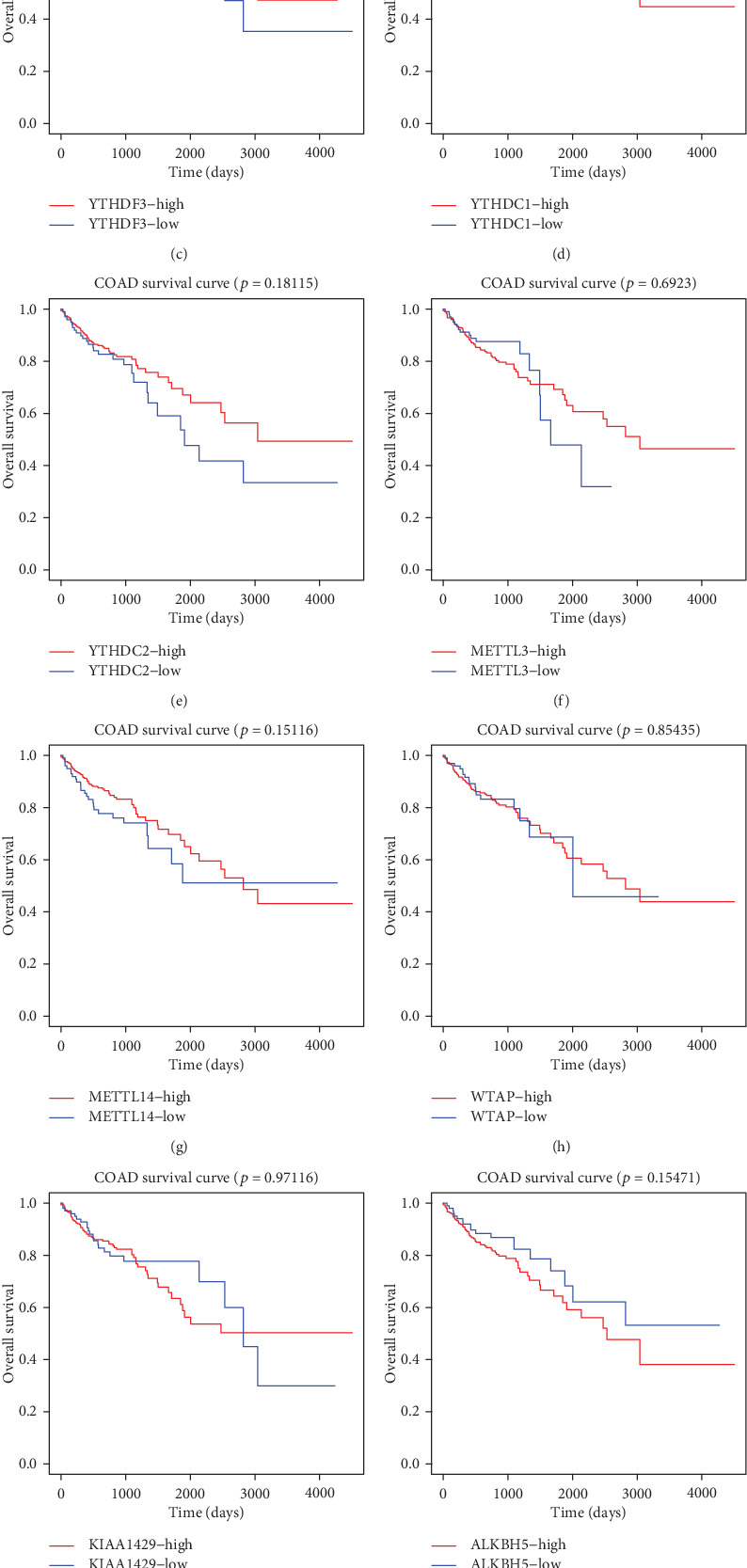
Correlation between expression levels of m^6^A RNA methylation regulators and overall survival rate. (a) YTHDF1; (b) YTHDF2; (c) YTHDF3; (d) YTHDC1; (e) YTHDC2; (f) METTL3; (g) METTL14; (g) WTAP; (i) KIAA1429; (j) ALKBH5; (k) FTO. The lower quartile of expression level was the cutoff point.

**Figure 5:**
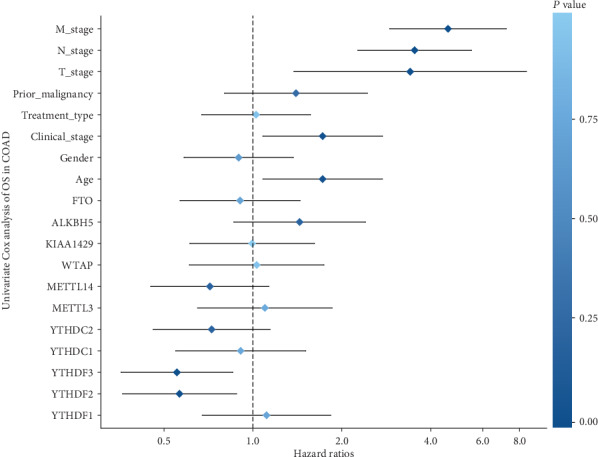
Cox regression analyses of the m^6^A RNA methylation regulators and clinicopathological factors in the 5-year overall survival of COAD. The horizontal axis shows the hazard ratio, and the vertical axis shows the predictive factors. M_stage: M1 vs. M0. N_stage: N2 vs. N0 or N1. Prior_malignancy: yes vs. no. Treatment_type: surgery vs. other. Clinical_stage: III or IV vs. I or II. Sex: male vs. female. Age: ≥65 vs. <65. All m^6^A RNA methylation regulators: high expression level vs. low expression level, where the cutoff value was the lower quartile of each regulator.

We used the lower quartile as the cutoff point to portray the survival curves of each m^6^A RNA methylation regulator. As shown in Figure 4, patients with the upregulated expression level of YTHDF2 and YTHDF3 had a better 5-year OS, with *HR* (95% CI) values of 0.564 (0.362–0.88) and 0.553 (0.358–0.854), respectively. Patients with higher T stage (*HR*: 3.409; 95% CI: 1.377-8.435; *P* = 0.008), M stage (*HR*: 3.529; 95% CI: 2.265-5.501; *P* < 0.001), and N stage (*HR*: 4.578; 95% CI: 2.907–7.209; *P* < 0.001) had a lower survival rate than patients with lower stages. We then performed a multivariate Cox regression analysis based on the selected indicators in the univariate Cox regression model (Figure 5) using the stepwise forward method. YTHDF3, together with age, N stage, and M stage, was an independent prognostic factor for COAD (Table 1).

### 3.4. Nomogram to Predict the Survival of COAD

**Figure 6:**
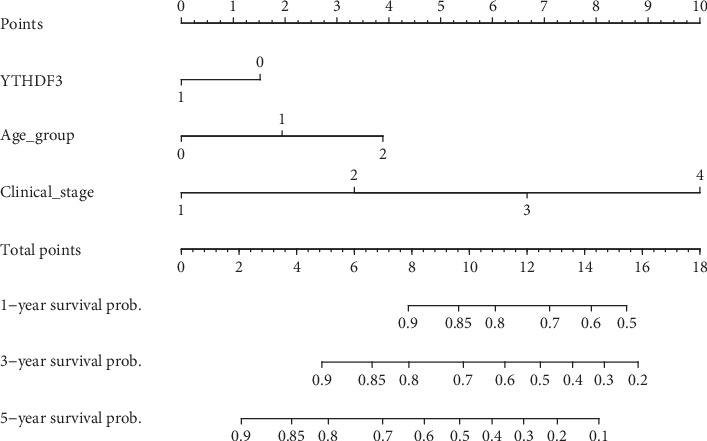
Nomogram used to predict the 1-year, 3-year, and 5-year survival probabilities of COAD patients. YTHDF3, 0: low expression level; 1: high expression level; the lower quartile is set as the cutoff value. Age group, 0: <65 years; 1: 65-79 years2: ≥80 years. Clinical_stage, 1-4 indicates stage I-IV.

Data on YTHDF3 expression, age, and clinical stages from TCGA dataset were used to establish a prognostic nomogram predicting overall survival based on the stepwise Cox regression model. Total score was obtained by adding up the individual contributions of the corresponding covariates on the points scale; the total score was used to predict 1-year, 3-year, and 5-year related survival probabilities (Figure 6).

### 3.5. GSEA Identified Related Signaling Pathways

Considering the critical associations of YTHDF1 with diagnosis, YTHDF3 with prognosis, and YTHDC2 with clinicopathological features observed by the above analysis, we further used the GSEA approach to explore the potential biological processes related to these genes. YTHDF1 mainly participates in basal transcription factors and spliceosomes (Supplementary Figure S1A and B). YTHDF3 is involved in ubiquitin-mediated proteolysis and the TGF-*β* signaling pathway (Supplementary Figure S1C and D). YTHDC2 is enriched in pathways of ubiquitin-mediated proteolysis, long-term potentiation, and prostate cancer (Supplementary Figure S1E, F, and G).

### 3.6. PPI Network Construction

Based on the STRING database, a coexpression network was constructed to explore interactions between m^6^A RNA methylation regulators (Supplementary Figure S2). We found that the writers (WTAP, KIAA1429, METTL3, and METTL14) closely interacted with each other (Supplementary Figure S2A). Further, we explored three module networks of selected m^6^A regulators (YTHDF1, YTHDF3, and YTHDC2) and broader genes. YTHDF1 was firmly related to YTHDF3 and shared many interacting proteins. Moreover, the writers (WTAP, KIAA1429, METTL3, and METTL14) also played essential roles in this network (Supplementary Figure S2B). In addition, many other genes were involved in forming the network, showing ample room for exploration.

## 4. Discussion

COAD is one of the most popular malignant gastrointestinal tract tumors and the second leading cause of cancer-related death in adults in Western countries [16]. During the past decades, many efforts have been made to search for biomarkers of COAD. Studies have shown the diagnostic value of DCTN1, DCTN2, and DCTN4 [17] and the prognostic role of LAYN [18], KCNQ1OT1 [19], and PYK2 [20] in COAD, but with limited sample size or inconsistent results. In this study, we analyzed the expression patterns of m^6^A RNA methylation regulators in COAD and their relationship with clinical features by using cohort data from TCGA. Most m^6^A-related proteins were dysregulated in COAD samples, and some were associated with clinical features. Among them, YTHDF1 had the highest diagnostic value for COAD. Survival analysis confirmed that high expression levels of YTHDF2 and YTHDF3 predicted a favorable prognosis. Moreover, YTHDF3 expression levels were independent prognostic factors of 5-year overall survival in COAD patients. YTHDC2 was highly expressed in female patients and showed a significant relationship with tumor progression. YTHDC1 was typically upregulated in younger patients. These results underscore the significant roles of the YTH domain family of readers, especially YTHDF1, YTHDF3, and YTHDC2, in the detection, progression, and prognosis of COAD.

In this study, we explored the roles of the YTH domain family of readers in COAD. m^6^A exerts many of its functions through reader proteins in the cytoplasm and nucleus that selectively bind to m^6^A-containing transcripts [21]. The "readers" are the bridge between "writers" and "erasers", and the ability of "readers" to block the accessibility of RNA demethylase may be critical to determining the m^6^A status of target transcripts [10], which may explain the critical roles of the YTH family in COAD. YTH domain family proteins include YTHDF1, YTHDF2, and YTHDF3 in the cytoplasm and YTHDC1 and YTHDC2 in the nucleus [22, 23]. YTHDF1 and YTHDF3 work in concert to affect the translation of m^6^A-containing mRNAs, YTHDF2 expedites mRNA decay, YTHDC1 affects the nuclear processing of its targets, and YTHDC2 selectively binds m^6^A at its consensus motif and enhances the translation efficiency of its targets and decreases their mRNA abundance [24–26]. Consistent with our results, Bai et al. reported that YTHDF1 could regulate tumorigenicity and cancer stem cell-like activity in colorectal carcinoma [27], and Tanabe et al. reported that YTHDC2 was upregulated in colon cancer and had a positive correlation with tumor stages [28]. Although YTHDF2, YTHDF3, and YTHDC1 showed essential roles in multiple cancer types [29–32], to date, no research on these regulators in COAD has been conducted. Therefore, the role of YTHDF2, YTHDF3, and YTHDC1 in COAD needs to be further studied. Our study showed a negative relationship between METTL14 and clinical stage, which implied its critical role in COAD progression. Ma et al. reported that METTL14 inhibits the metastatic potential of hepatocellular carcinoma by modulating N6-methyladenosine-dependent primary microRNA processing [33]. Further research is needed to explore the function of METTL14.

Moreover, our study showed a high correlation between the expression of YTHDF3 and KIAA1429 in COAD. We also observed a moderate correlation between METTL14 and YTHDF2, YTHDC1, and YTHDC2. The PPI network showed the interactions among "writers", "readers", and "erasers". The cross-talk among different categories of m^6^A regulators may influence cancer growth and progression. Chen et al. found that METTL3 promoted liver cancer progression through YTHDF2-dependent posttranscriptional silencing of SOCS2 [34]. Panneerdoss et al. reported that METTL14 and ALKBH5 inhibited the expression of YTHDF3, reversely block RNA demethylase activity, and altered the m^6^A status of target transcripts in cancer cells [10]. These studies have emphasized interactions between writers, readers, and erasers. Moreover, the interactions of members in the same category are also frequent. For example, YTHDF3 initiates mRNA translation and methylated mRNA decay through cooperation with YTHDF1 and YTHDF2, affecting the functions of the other two YTHDFs [25]. Therefore, the interactions of these regulators may play essential roles in COAD and should be further explored.

GSEA revealed that YTHDF1, YTHDF3, and YTHDC2 were enriched in several meaningful pathways. YTHDF3 and YTHDC2 share the same pathways of ubiquitin-mediated proteolysis, which further illustrates their functional biological connection. Taniue et al. found that lncRNA UPAT and UHRF1 may be promising molecular targets for the therapy of colon cancer by regulating protein ubiquitination and degradation and thereby play a critical role in the survival and tumorigenicity of cancer cells [35]. We suggest that the ubiquitin-mediated proteolysis affected by YTHDF3 and YTHDC2 may play a crucial role in tumor progression. The spliceosome can be induced for the activation of splicing and mRNA production in the carcinogenic process. Takayama et al. suggested that targeting spliceosome proteins by RNA-binding protein to promote AR splicing and expression could be a therapeutic possibility for hormone-refractory prostate cancer [36]. We hypothesize that YTHDF1 could also be a potential therapeutic target by modulating spliceosome and other RNA activities. TGF-*β* promotes tumor growth and metastasis by inducing angiogenic factors and facilitating EMT [37]. TGF-*β* signaling-associated genes are particularly sensitive to changes in m^6^A levels [10]; thus, the malfunction of the TGF-*β* signaling pathway in response to YTHDF3 dysregulation may be the main factor affecting the prognosis of COAD.

## 5. Conclusions

Most m^6^A-related proteins are dysregulated in COAD samples compared to normal samples, and some are associated with clinical features. Our results indicated a significant role of the YTH domain family ("readers") in the diagnosis, progression, and prognosis of COAD. The exact mechanism of the YTH domain family of N6-methyladenosine readers is worth further study.

## Figures and Tables

**Table 1:** Multivariate Cox regression analysis of selected prognostic factors in the overall survival of COAD patients.

Factors	*P* value	HR (95% CI)^†^
M stage (M1 vs. M0)	<0.001	3.51 (2.13-5.76)
N stage (N2 vs. N0 or N1)	<0.001	2.36 (1.46-3.84)
YTHDF3 (high level vs. low level)	0.015	0.58 (0.37-0.90)
Age (≥65 years vs. <65 years)	0.014	1.81 (1.13-2.89)

^†^HR: hazard ratio; CI: confidence interval; the lower quartile was the cutoff value for YTHDF3.

## Data Availability

All data generated or analyzed during this study are included in this published article.

## Supplementary Materials

Supplementary Materials Supplementary Figure S1: Pathways enriched according to GSEA of YTHDF1, YTHDF3, and YTHDC2. Supplementary Figure S2: PPI network of m^6^A RNA methylation regulators.
