# Supplementary material for: The YTH Domain Family of N6-Methyladenosine “Readers” in the Diagnosis and Prognosis of Colonic Adenocarcinoma
Source: Biomed Res Int. 2020 May 30;2020:9502560. doi: 10.1155/2020/9502560 (PMC7277069; doi:10.1155/2020/9502560)
Supplement: Supplementary Materials — Supplementary Figure S1: Pathways enriched according to GSEA of YTHDF1, YTHDF3, and YTHDC2. Supplementary Figure S2: PPI network of m6A RNA methylation regulators. [file 9502560.f1.zip › 9502560.f1/mat.9502560.v2--revised.pdf]

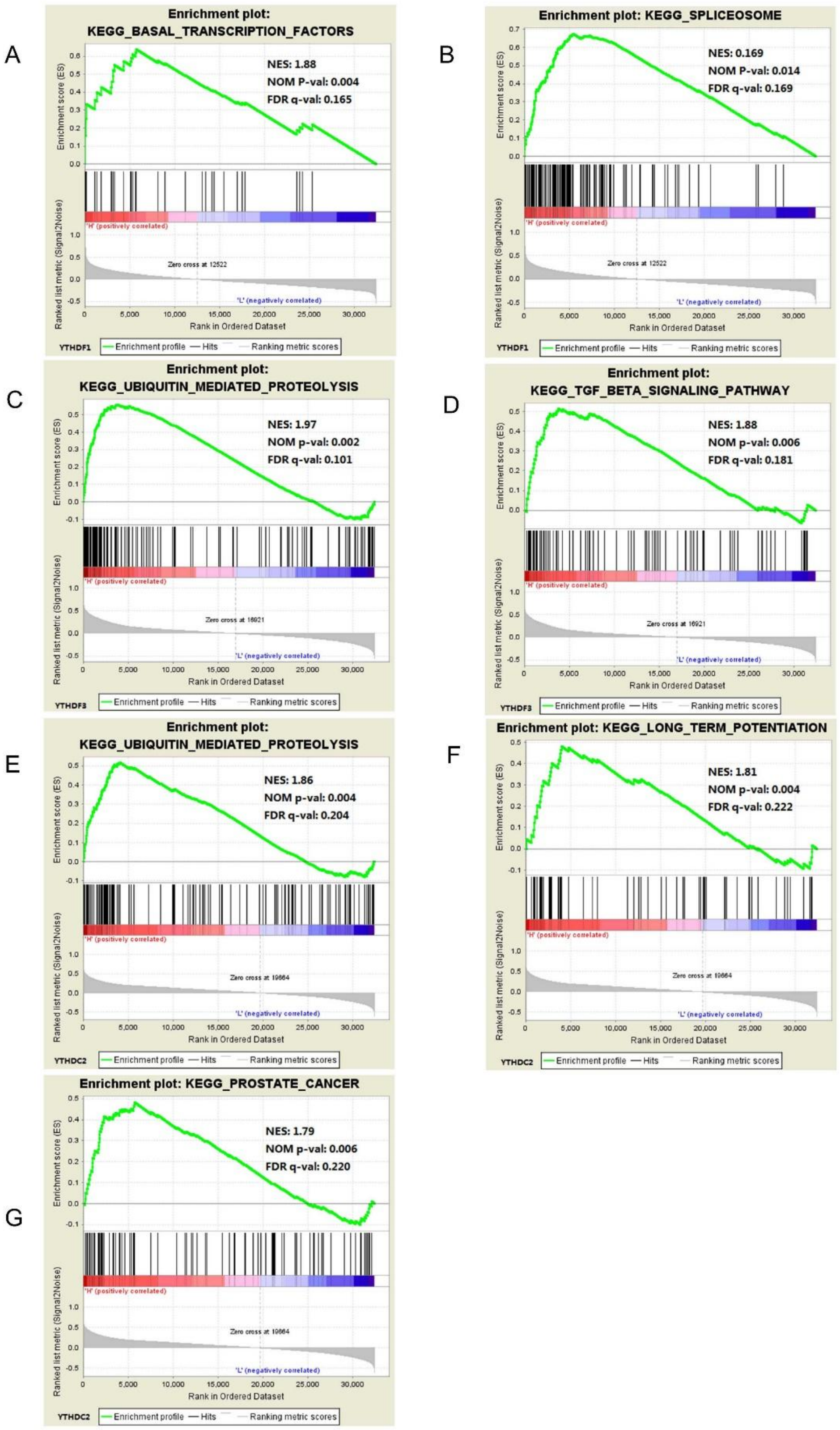

Supplementary Figure 1. Pathways enriched by GSEA analysis on YTHDF1, YTHDF3, and YTHDC2.

A

B

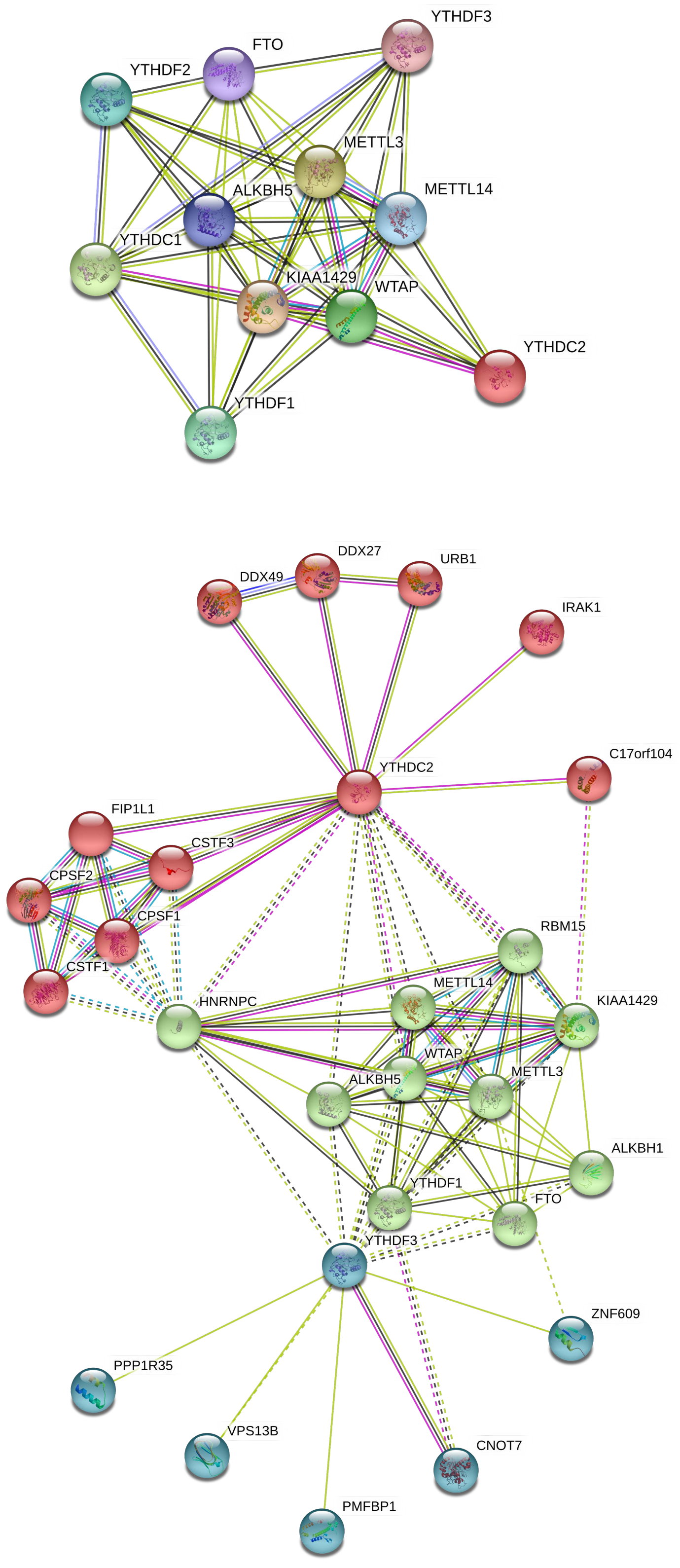

479

480 **Supplementary Figure 2.** PPI network of m^6A RNA methylation regulators.
