# Supplementary material for: The YTH Domain Family of N6-Methyladenosine “Readers” in the Diagnosis and Prognosis of Colonic Adenocarcinoma
Source: Biomed Res Int. 2020 May 30;2020:9502560. doi: 10.1155/2020/9502560 (PMC7277069; doi:10.1155/2020/9502560)
Supplement: Supplementary Materials — Supplementary Figure S1: Pathways enriched according to GSEA of YTHDF1, YTHDF3, and YTHDC2. Supplementary Figure S2: PPI network of m6A RNA methylation regulators. [file 9502560.f1.zip › 9502560.f1/Supplementary Figure S1_BMRI_3081521.pdf]

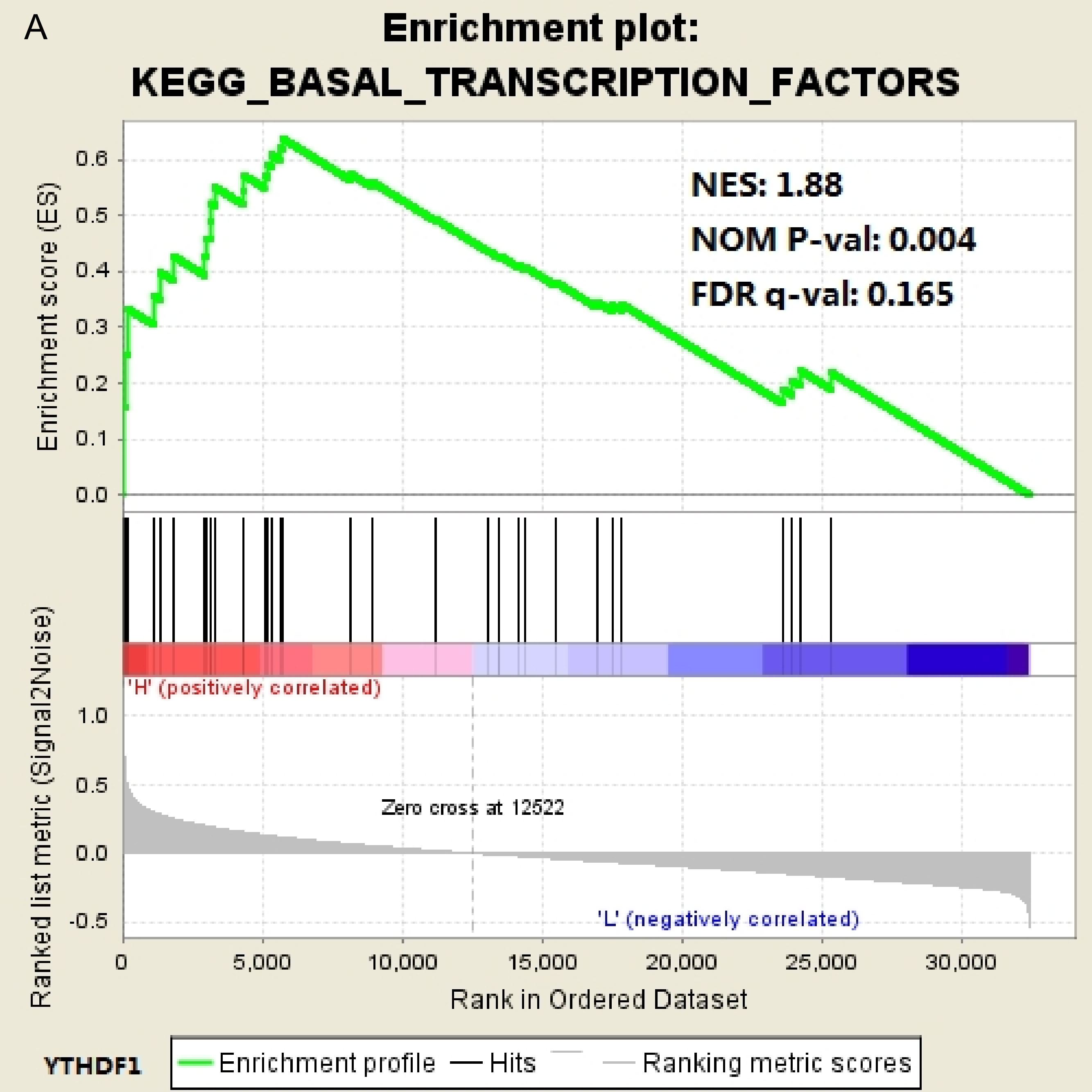
A
Enrichment plot:
KEGG_BASAL_TRANSCRIPTION_FACTORS
NES: 1.88
NOM P-val: 0.004
FDR q-val: 0.165
Enrichment score (ES)
0.6
0.5
0.4
0.3
0.2
0.1
0.0
'H' (positively correlated)
Zero cross at 12522
'L' (negatively correlated)
Ranked list metric (Signal2Noise)
1.0
0.5
0.0
-0.5
0
5,000
10,000
15,000
20,000
25,000
30,000
Rank in Ordered Dataset
YTHDF1
Enrichment profile
Hits
Ranking metric scores

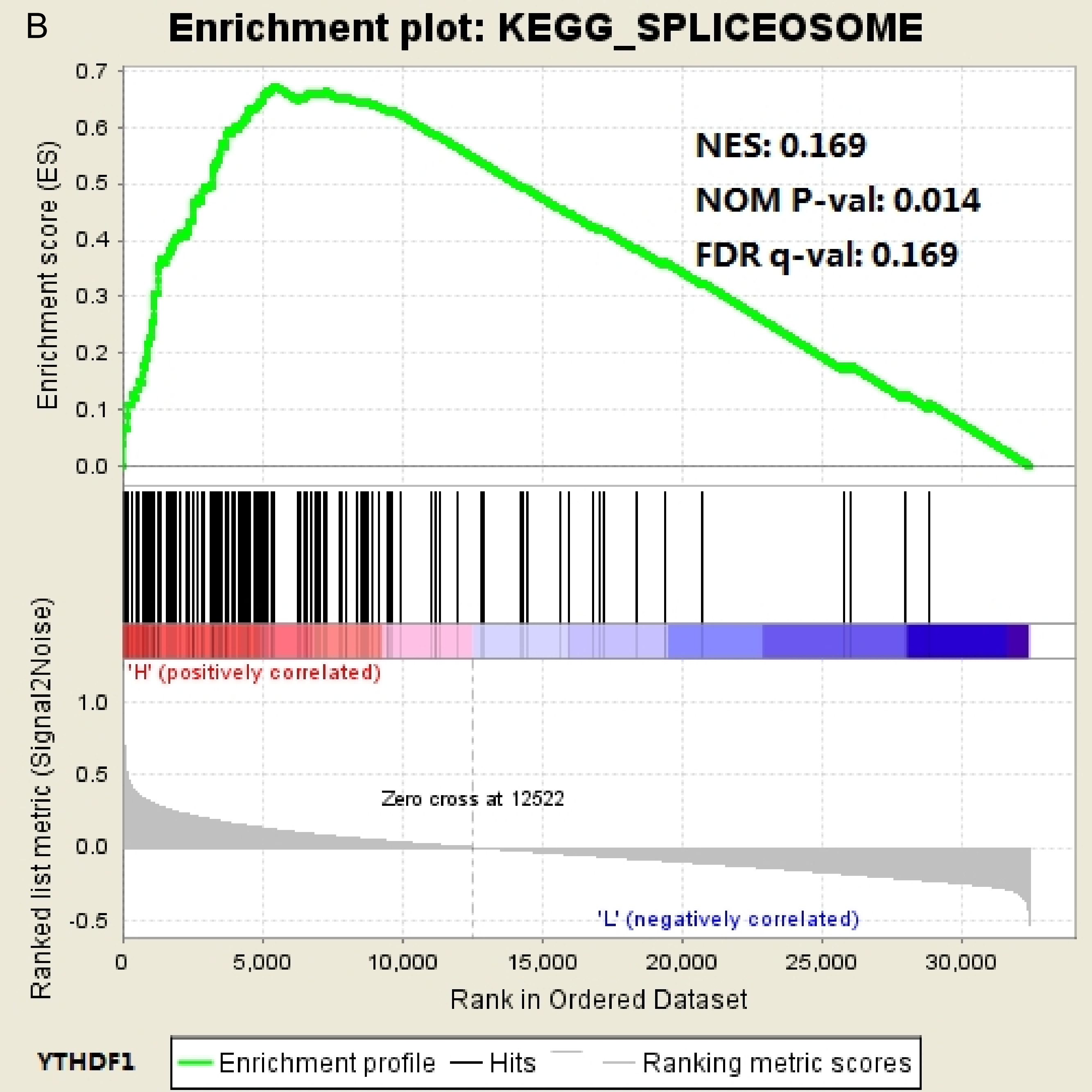

B
Enrichment plot: KEGG_SPLICEOSOME
NES: 0.169
NOM P-val: 0.014
FDR q-val: 0.169
Enrichment score (ES)
'H' (positively correlated)
Zero cross at 12522
'L' (negatively correlated)
Ranked list metric (Signal2Noise)
Rank in Ordered Dataset
YTHDF1
Enrichment profile
Hits
Ranking metric scores

C

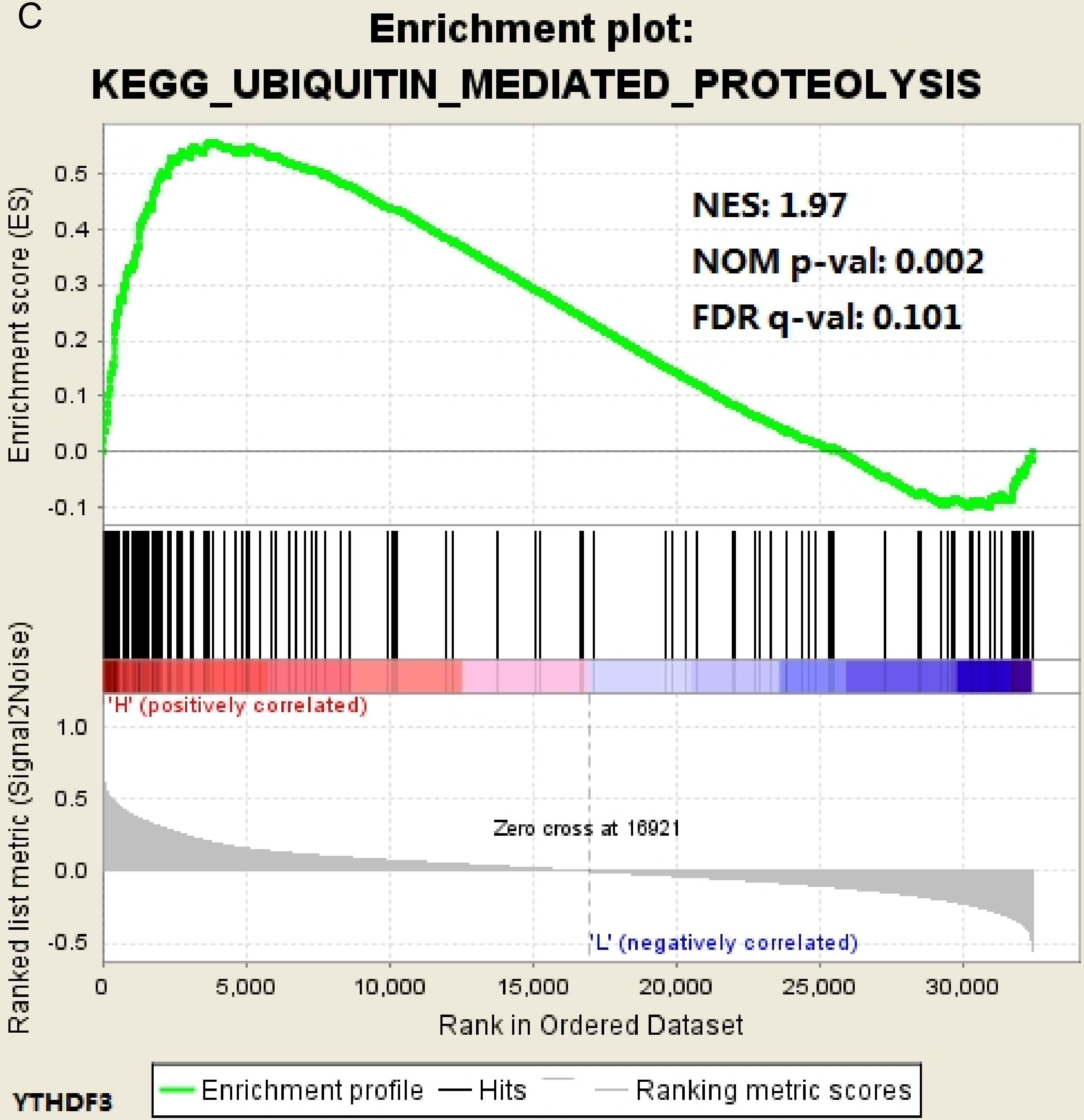

Enrichment plot:
KEGG_UBIQUITIN_MEDIATED_PROTEOLYSIS
NES: 1.97
NOM p-val: 0.002
FDR q-val: 0.101
Enrichment score (ES)
0.5
0.4
0.3
0.2
0.1
0.0
-0.1
'H' (positively correlated)
Ranked list metric (Signal2Noise)
1.0
0.5
0.0
-0.5
Zero cross at 16921
'L' (negatively correlated)
0
5,000
10,000
15,000
20,000
25,000
30,000
Rank in Ordered Dataset
Enrichment profile
Hits
Ranking metric scores
YTHDF3

D

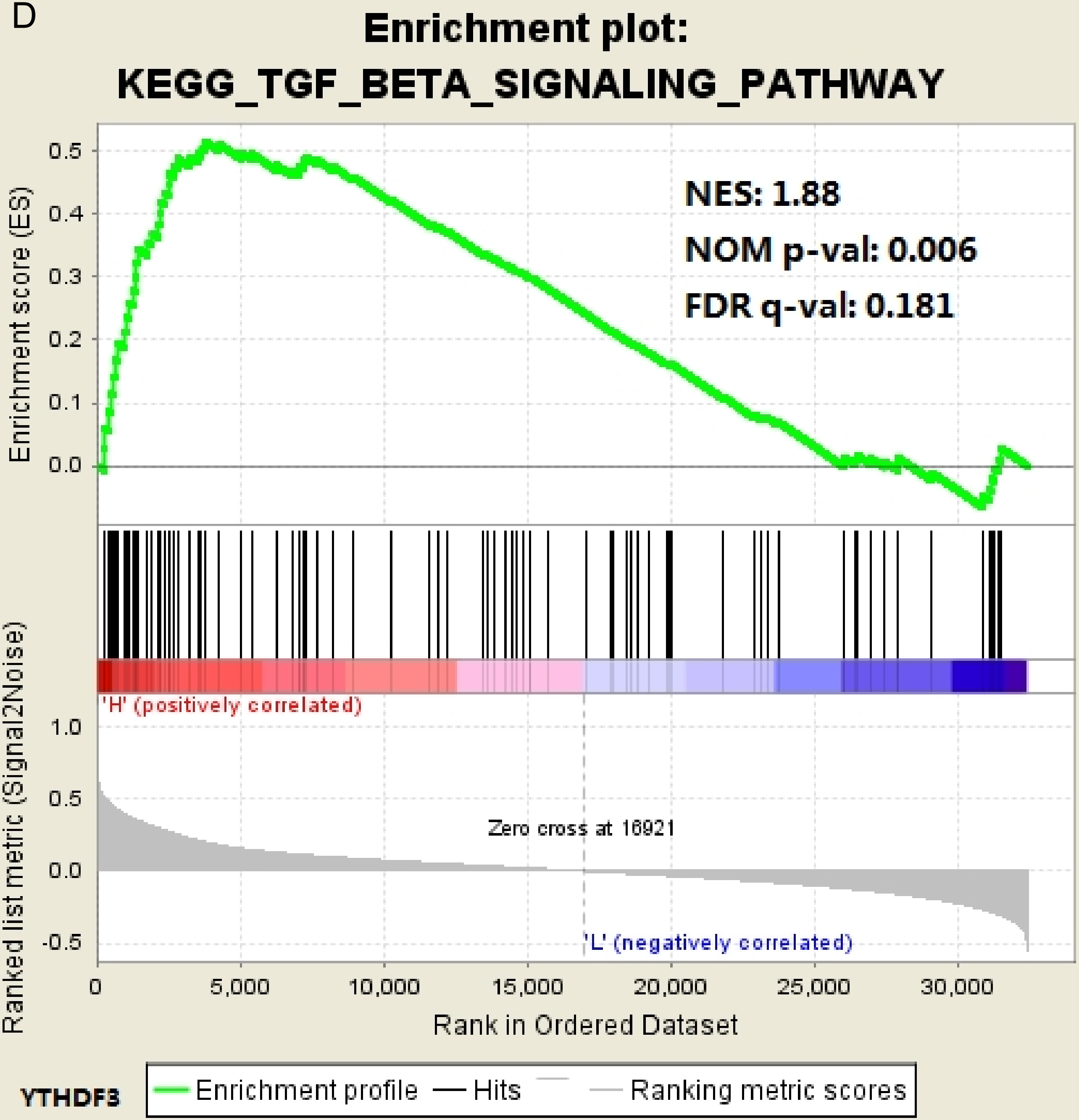
Enrichment plot:
KEGG_TGF_BETA_SIGNALING_PATHWAY
NES: 1.88
NOM p-val: 0.006
FDR q-val: 0.181
Enrichment score (ES)
0.5
0.4
0.3
0.2
0.1
0.0
'H' (positively correlated)
Zero cross at 16921
'L' (negatively correlated)
Ranked list metric (Signal2Noise)
1.0
0.5
0.0
-0.5
0
5,000
10,000
15,000
20,000
25,000
30,000
Rank in Ordered Dataset
Enrichment profile
Hits
Ranking metric scores
YTHDF3

E

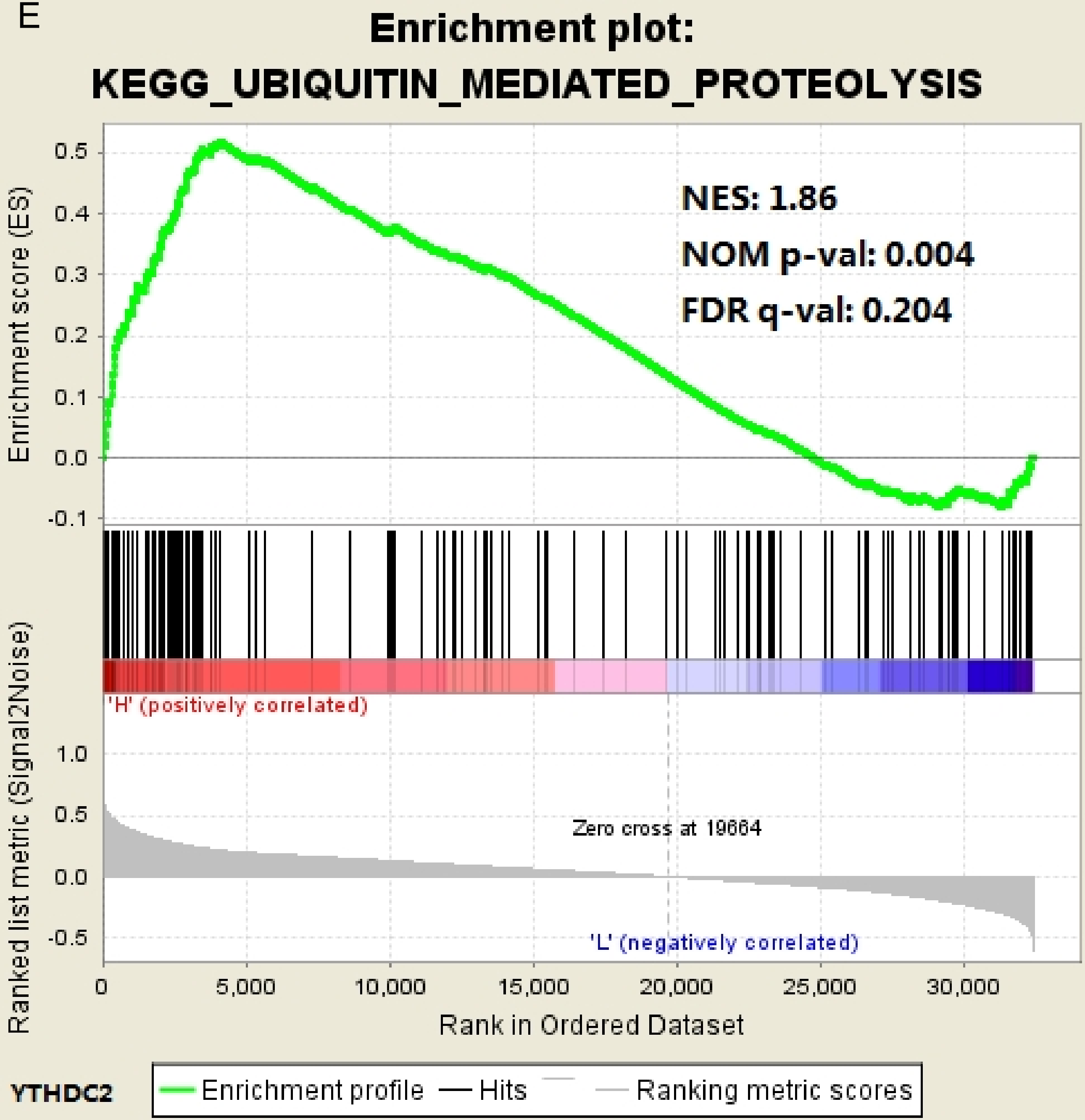

Enrichment plot:
KEGG_UBIQUITIN_MEDIATED_PROTEOLYSIS
NES: 1.86
NOM p-val: 0.004
FDR q-val: 0.204
Enrichment score (ES)
0.5
0.4
0.3
0.2
0.1
0.0
-0.1
'H' (positively correlated)
Ranked list metric (Signal2Noise)
1.0
0.5
0.0
-0.5
Zero cross at 19664
'L' (negatively correlated)
0
5,000
10,000
15,000
20,000
25,000
30,000
Rank in Ordered Dataset
YTHDC2
Enrichment profile
Hits
Ranking metric scores

F

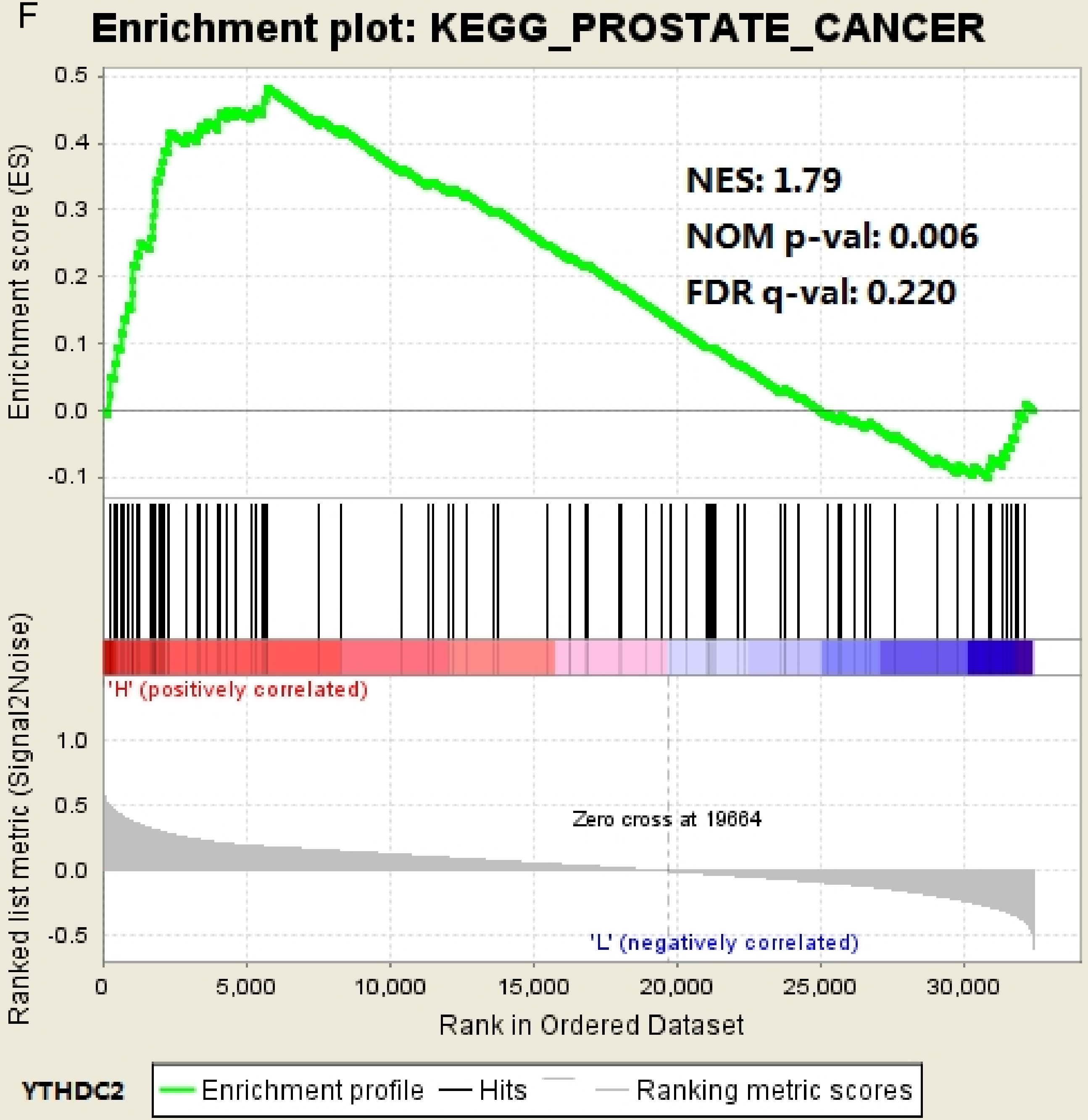

Enrichment plot: KEGG_PROSTATE_CANCER
NES: 1.79
NOM p-val: 0.006
FDR q-val: 0.220
Enrichment score (ES)
0.5
0.4
0.3
0.2
0.1
0.0
-0.1
'H' (positively correlated)
Zero cross at 19664
'L' (negatively correlated)
Ranked list metric (Signal2Noise)
1.0
0.5
0.0
-0.5
0
5,000
10,000
15,000
20,000
25,000
30,000
Rank in Ordered Dataset
YTHDC2
Enrichment profile
Hits
Ranking metric scores

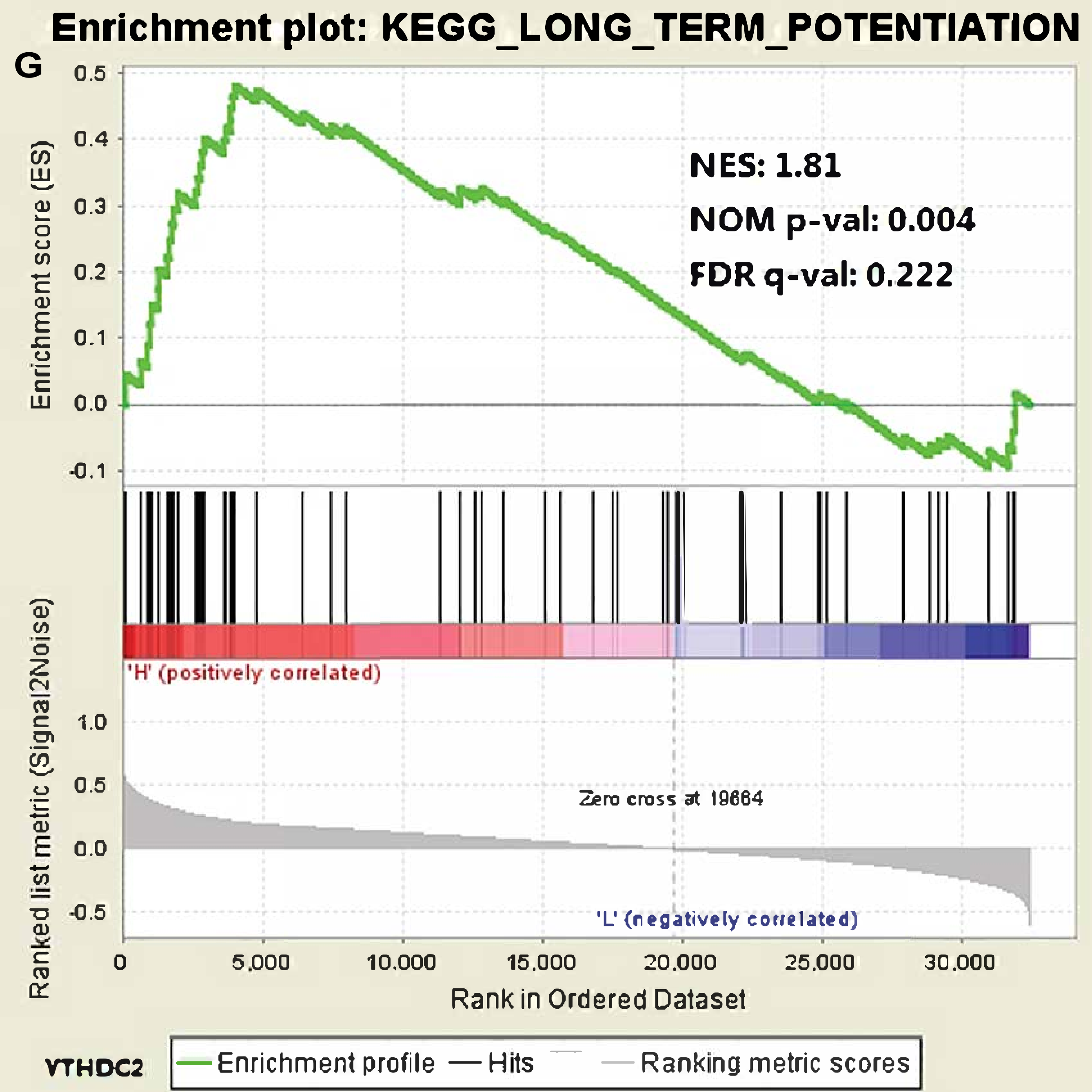

Enrichment plot: KEGG_LONG_TERM_POTENTIATION
G
Enrichment score (ES)
0.5
0.4
0.3
0.2
0.1
0.0
-0.1
NES: 1.81
NOM p-val: 0.004
FDR q-val: 0.222
'H' (positively correlated)
Ranked list metric (Signal2Noise)
1.0
0.5
0.0
-0.5
Zero cross at
'L' (negatively correlated)
0
5,000
10,000
15,000
20,000
25,000
30,000
Rank in Ordered Dataset
YTHDC2
Enrichment profile
Hits
Ranking metric scores
