# Supplementary figures and images for: The YTH Domain Family of N6-Methyladenosine “Readers” in the Diagnosis and Prognosis of Colonic Adenocarcinoma
Source: Biomed Res Int. 2020 May 30;2020:9502560. doi: 10.1155/2020/9502560 (PMC7277069; doi:10.1155/2020/9502560)

A

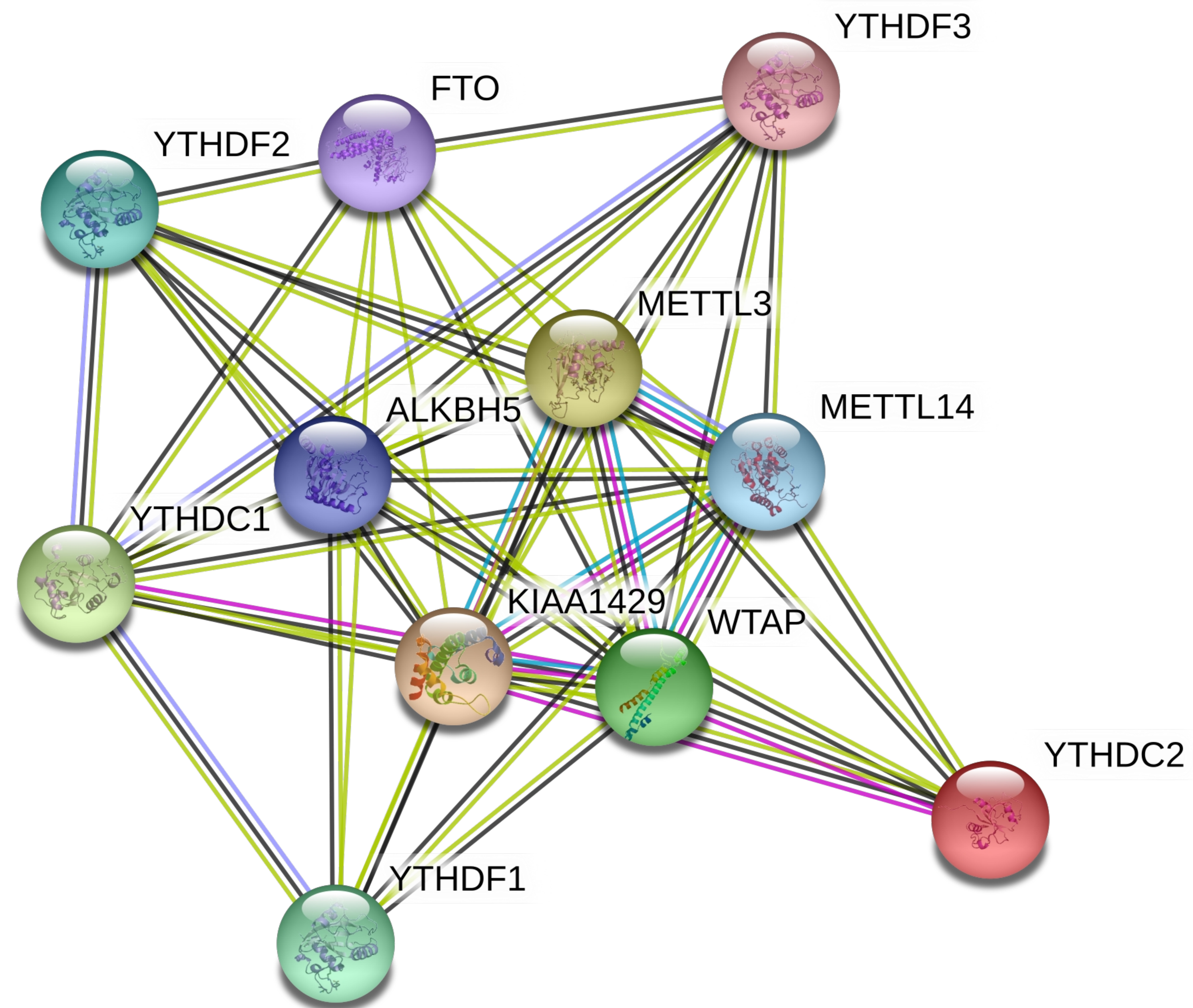

B

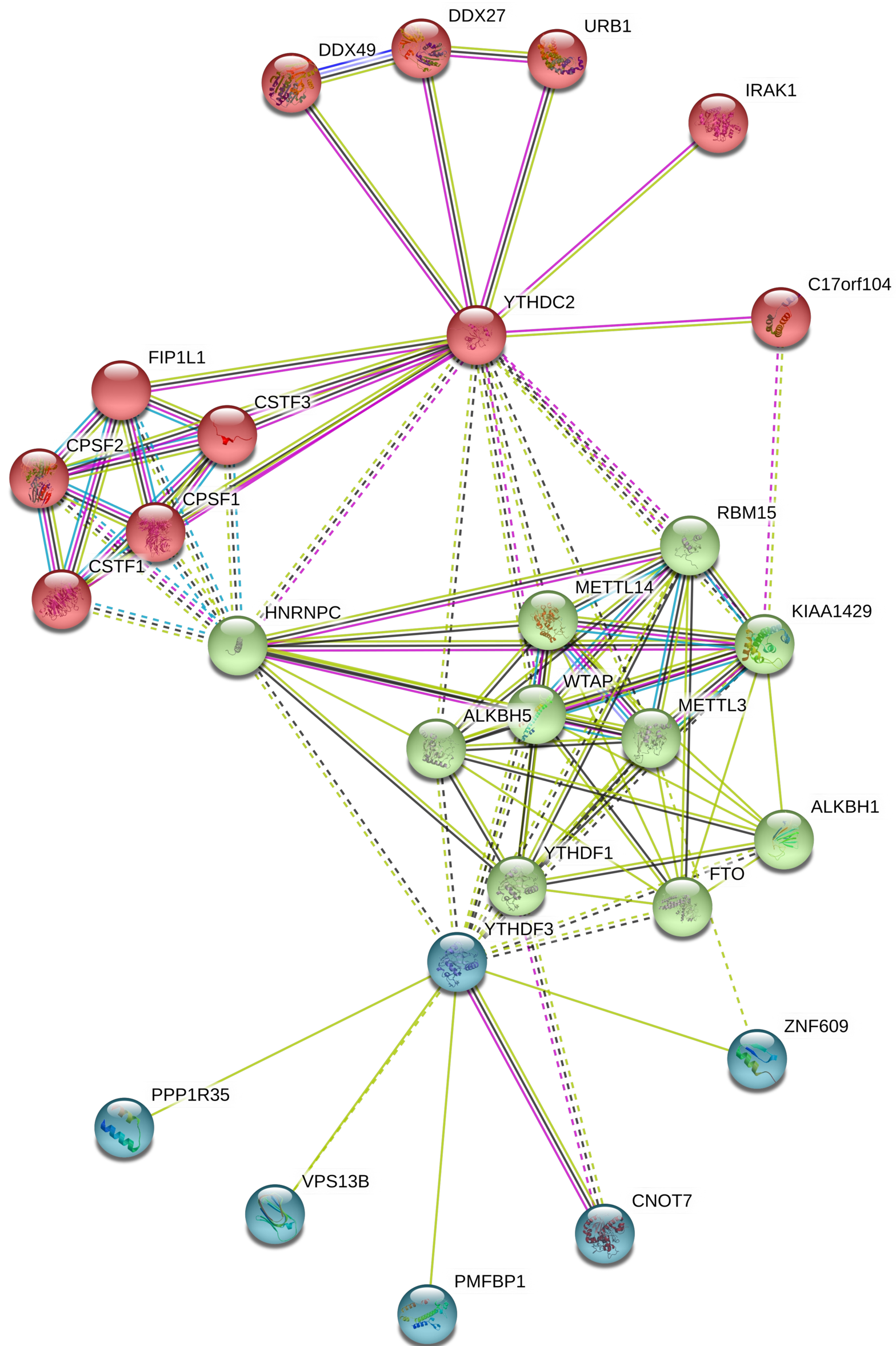

Supplement: Supplementary Materials — Supplementary Figure S1: Pathways enriched according to GSEA of YTHDF1, YTHDF3, and YTHDC2. Supplementary Figure S2: PPI network of m6A RNA methylation regulators. [file 9502560.f1.zip › 9502560.f1/Supplementary Figure S2_BMRI_3081522.pdf]
